# Similar regulatory mechanisms of caveolins and cavins by myocardin family coactivators in arterial and bladder smooth muscle

**DOI:** 10.1371/journal.pone.0176759

**Published:** 2017-05-25

**Authors:** Baoyi Zhu, Catarina Rippe, Tran Thi Hien, Jianwen Zeng, Sebastian Albinsson, Karin G. Stenkula, Bengt Uvelius, Karl Swärd

**Affiliations:** 1 Department of Experimental Medical Science, Lund University, Lund, Sweden; 2 Department of Urology, the Sixth Affiliated Hospital of Guangzhou Medical University, Guangdong, China; 3 Department of Urology, Clinical Sciences, Lund University, Lund, Sweden; University of British Columbia, CANADA

## Abstract

Caveolae are membrane invaginations present at high densities in muscle and fat. Recent work has demonstrated that myocardin family coactivators (MYOCD, MKL1), which are important for contractile differentiation and cell motility, increase caveolin (CAV1, CAV2, CAV3) and cavin (CAVIN1, CAVIN2, CAVIN3) transcription, but several aspects of this control mechanism remain to be investigated. Here, using promoter reporter assays we found that both MKL1/MRTF-A and MKL2/MRTF-B control caveolins and cavins via their proximal promoter sequences. Silencing of MKL1 and MKL2 in smooth muscle cells moreover reduced CAV1 and CAVIN1 mRNA levels by well over 50%, as did treatment with second generation inhibitors of MKL activity. GATA6, which modulates expression of smooth muscle-specific genes, reduced CAV1 and CAV2, whereas the cavins were unaffected or increased. Viral overexpression of MKL1 and myocardin induced caveolin and cavin expression in bladder smooth muscle cells from rats and humans and MYOCD correlated tightly with CAV1 and CAVIN1 in human bladder specimens. A recently described activator of MKL-driven transcription (ISX) failed to induce CAV1/CAVIN1 which may be due to an unusual transactivation mechanism. In all, these findings further support the view that myocardin family coactivators are important transcriptional drivers of caveolins and cavins in smooth muscle.

## Introduction

Caveolae are omega-formed organelles present in e.g. muscle, endothelia and adipocytes [1]. Formation of caveolae depends on caveolins (CAV1-CAV3) and cavins (CAVIN1, CAVIN2, CAVIN3, CAVIN4) and mutations that cause loss of caveolae give rise to muscular dystrophy, lipodystrophy and cardiac rhythm disturbances [2–5]. CAV1 and CAV3 can substitute for each other in formation of caveolae, but these proteins have distinct expression profiles due to differing transcriptional control mechanisms. If one aspires to pharmacologically control the expression of caveolins and cavins to treat e.g. cavolinopathies [6], it is necessary to understand the genetic control mechanisms that govern tissue-specific expression of caveolins and cavins. Insight into the transcriptional machinery of these proteins may also be helpful in regenerative medicine, when stem cells are guided to adopt a mature and functional cell fate for organ reconstruction.

Advances have been made with regard to transcriptional regulation of caveolins and cavins, but the picture is far from complete. Early work indicated the presence of sterol regulatory elements in the promoter of CAV1 [7], conferring sensitivity to cholesterol loading. CAV1 has also been demonstrated to be under control of PPARγ signaling [8]. Other transcriptional control mechanisms for CAV1 include FOXO transcription factors [9] and EGR1. EGR1 is suppressive and its inhibition is relieved by mechanical stimulation [10]. In addition, CAV1 is reportedly increased by the hypoxia-inducible factor [11], which also inhibits cavin-1 (CAVIN1) and cavin-2 (CAVIN2) expression [12]. Work in bladder smooth muscle moreover demonstrated that CAV1 expression is inhibited by GATA6 [13], but whether cavins are similarly controlled has not be examined.

Recent work by us demonstrated that two myocardin family coactivators (MYOCD and MKL1/MRTF-A) stimulate biogenesis of caveolae by increasing the mRNA levels of caveolins and cavins in coronary artery smooth muscle cells (SMCs). Myocardin family coactivators appear unique in that they induce essentially all caveolins and cavins [14]. Several questions remain unanswered with respect to this transcriptional control mechanism, however [4]. For example, the myocardin family has four members (MYOCD, MKL1/MRTF-A, MKL2/MRTF-B, MAMSTR), and it is not known whether MKL2 and MAMSTR control caveolin and cavin expression in smooth muscle similar to MKL1 and MYOCD. The DNA elements responsible for MKL-driven caveolin and cavin expression moreover remain poorly defined, and only in the case of CAV1 has the proximal promoter been implicated. Work with actin depolymerizing agents and a small molecule MKL/MRTF inhibitor supported involvement of MKLs in biogenesis of caveolae in coronary artery SMCs [14], but it is not known if the myocardin family drives caveolae in other smooth muscles or indeed in any other tissue.

In the present study we have addressed a number of questions regarding the control of caveolins and cavins by myocardin family coactivators in smooth muscle from urinary bladder, coronary arteries, and aorta, and from several species, including man. Using promoter reporter assays we show that MKL1 and MKL2 regulate caveolins and cavins via proximal promoter sequences in all cases. We also demonstrate that siRNA-mediated silencing of MKL1 and MKL2 reduces CAV1/CAVIN1 mRNA levels by 50–70%, and using first and second generation MKL inhibitors we show sizeable repression of CAV1/CAVIN1 in bladder smooth muscle from rats and humans. These findings further support the notion [14] that myocardin family coactivators constitute an important genetic control mechanism for formation of caveolae in smooth muscle.

## Materials and methods

### Ethics statements

Protocols for mice and rats were approved by the Malmö/Lund Ethical Committee for animal experiments (approval numbers M 4–16 and M 46–13), and animal handling conformed to national guidelines and the Directive 2010/63/EU of the European Parliament. Animals were sacrificed using ascending CO_2_ in air. Human detrusor muscle was retrieved from cystectomized bladders following written informed consent [15] and after approval from the Regional Ethical Review Board at Lund University (http://www.epn.se, approval number 2008–4) and the Declaration of Helsinki. Coronary artery SMCs of human origin were from Gibco (Life Technologies) who guarantees that cells are obtained with informed consent.

### Animals

Female Sprague-Dawley rats (200 g) and C57BL/6 mice (25 g) of both sexes were used. Animals were housed at the conventional animal facility at BMC, Lund, and maintained on a 12h light/dark cycle. Health was monitored using FELASA guidelines and animals were fed standard chow ad libitum.

### Cell culture and adenoviral transduction

SMCs were isolated from rat bladders and the mouse aorta by enzymatic digestion and maintained in culture essentially as described previously [16–18]. For SMC isolation, loosely attached tissue and the mucosa were aseptically removed from the smooth muscle layer under a dissection microscope and in cold physiological buffer. The remaining muscle layer was cut into fine pieces. The pieces were digested in serum-free DMEM cell culture medium (Gibco, catalog no. 11966-025) containing 2 mg/ml collagenase type 2 (Worthington Biochemical Corporation) and 0.2 mg/ml elastase (Sigma) under standard culture conditions (37°C in a humidified atmosphere of 95% air/5% CO_2_) for 3 hours with gentle manual tumbling every 30 min. Remaining tissue pieces were allowed to sediment, and the supernatant was aspirated and centrifuged at 1000 RPM for 3 min. The resulting cell pellet was then suspended in DMEM/Ham's F-12 medium with stable glutamine (Biochrom, FG4815), 10% fetal bovine serum (FBS) (Biochrom, catalog no. S0115) and 50μg/ml penicillin/streptomycin (Biochrom, catalog no. A 2212). The medium was exchanged every 48 h. SMCs between passages 5 to 8 were used in all experiments.

### Viral transduction

Adenoviruses, Ad-CMV-MKL1 (MRTF-A), Ad-CMV-MKL2 (MRTF-B), Ad-CMV-MYOCD, Ad-CMV-GATA6 and Ad-CMV-null, were from Vector Biolabs. For overexpression, bladder SMCs were transduced using 100 MOI (multiplicity of infection) 24h after seeding. Cells were then maintained in virus-containing media for 24h and for another 48h in fresh DMEM/Ham's F-12 with 10% FBS before harvest. Ad-CMV-null (Vector Biolabs) was used as control throughout.

### Reporter assays

Promoter reporters were from Switchgear genomics (Active Motif, Belgium) and encompassed on average 1000 nucleotides 5’ of the transcription initiation site (CAV1, 1077nt; CAV2, 957nt; CAV3, 1033 nt; CAVIN1, 928nt; CAVIN2, 1263nt; CAVIN3, 988nt). Human coronary artery SMCs on 6-well plates were grown in Medium 231 (Life Technologies) with 5% smooth muscle growth supplement (Life Technologies, M-231-500) and 50U/50μg/ml penicillin/streptomycin (Biochrom, A 2212) in a standard cell culture incubator as described above. Cells were transduced with Ad-CMV-MKL1 (MRTF-A), Ad-CMV-MKL2 (MRTF-B) or Ad-CMV-null, and after 24h they were transfected with promoter reporters using FuGENE 6 Transfection Reagent (Promega, cat E2691). Cells were frozen at 96h followed by lysis in 100μl reporter lysis buffer (Promega). Luciferase activity was determined in a Glomax instrument (Promega) using the LightSwith Luciferase Assay System (Active Motif, Belgium).

### Gene silencing

Silencer Select predesigned siRNAs (catalog no. 4390771, Thermo Scientific, 20 nM MKL1 siRNA (s73019), 20 nM MKL2 siRNA (s109074), or 20 nM control siRNA) were transfected using Oligofectamine transfection reagent (Invitrogen) as described [16]. Cells were harvested at 96h for RNA isolation.

### RNA isolation and qRT-PCR

Cells were washed twice with ice-cold phosphate buffered saline (PBS, Biochrom) and lysed in Qiazol (Qiagen, cat. 79306). Total RNA was obtained using the Qiagen miRNeasy mini kit (Qiagen #217004). RNA purity and concentration was assessed using a Nanodrop spectrophotometer (Thermo Scientific). PCR reactions were run with the Quantifast SYBR Green RT-PCR kit (Qiagen, #204156) and Quantitect (Qiagen) primer assays for CAV1, CAV2, CAV3, CAVIN1, CAVIN2, CAVIN3, CNN1, GATA6, MKL1 and MKL2. As a reference gene we used 18S throughout. Primer sequences are proprietary information of Qiagen. PCR reactions were run in a real time thermal cycler (StepOnePlus, Applied Biosystems).

### Pharmacological treatment

Rat and human bladder SMCs were transduced using 100 MOI of Ad-CMV-MRTF-A or Ad-CMV-null 24h after seeding. Following incubation in virus-containing media for 24h, cells were starved in DMEM/Ham's F-12 medium with 2% FBS for another 24h. N-cyclopropyl-5-(thiophen-2-yl)-isoxazole-3-carboxamide (ISX, 20μM) or the equivalent volume of DMSO was then added and cells were harvested after another 48h. The effects of CCG-1423 (Cayman Chemical Company, 10010350), CCG-100602 (Cayman Chemical Company, 10787) and CCG-203971 (Tocris Bioscience, 5277), which inhibit MKL/SRF signaling, were tested in serum starved cells. 24h after seeding, rat and human bladder SMCs were starved in DMEM/Ham's F-12 medium with 2% FBS for 24h. CCG-1423, CCG-100206, CCG203971, all at 10 μM, or the equivalent volume of DMSO (Sigma Aldrich, D5879) was then added. The cells were harvested after another 24h.

### Protein extraction and western blotting

Cells were washed using ice-cold PBS and lysed in 70 μl of 1X Laemmli sample buffer (60 mM Tris-Hcl, pH 6.8, 10% glycerol, 2% SDS). Protein determination was performed using the Biorad DC protein assay and 0.01% bromophenol and 5% β-mercaptoethanol were added. 20μg protein was loaded per lane on TGX 4–15% Criterion gels (Bio-Rad, no. 567-1084). Following 1D separation and transfer to nitrocellulose, proteins were detected using the following antibodies: CAV1 (Cell Signaling, cat. D46G3), CAV2 (BD Transduction Laboratories, 610685), CAV3 (BD Transduction Laboratories 610421), CAVIN11 (Abcam ab48824), CAVIN2 (Abcam, ab113876), CAVIN3 (Proteintech, 16250-1-AP), MKL1/MRTF-A (Cell Signaling Technology, #14760), CNN1 (Abcam, ab46794), HSP90 (BD Transduction Laboratories, 610418). Primary antibodies were used at dilutions recommended by the vendors. Secondary HRP-conjugated anti mouse or anti rabbit antibodies (Cell Signaling) were used for detection with SuperSignal West Pico Chemiluminescent Substrate (Pierce, cat34080) in an Odyssey Fc Imager (LI-COR Biosciences). Image Studio 3.1 was used for image capture and analysis.

### Statistical analysis

Results are presented as means±S.E.M. For single statistical comparisons a two-tailed student's t-test for paired or unpaired data was used. For multiple comparisons a one-way ANOVA followed by the Bonferroni post-hoc test was used. GTEx expression data was downloaded and TMM normalized as described [14], and correlations were tested using the Spearman method. All statistical computations were made using Prism/InStat (GraphPad Prism 5.0 Software) using log-transformed data. N indicates the number of preparations. A minimum of three samples were included in each group. P<0.05 was considered significant. Significance is indicated by *P<0.05, **P<0.01, and ***P<0.001.

## Results

**Fig 1:**
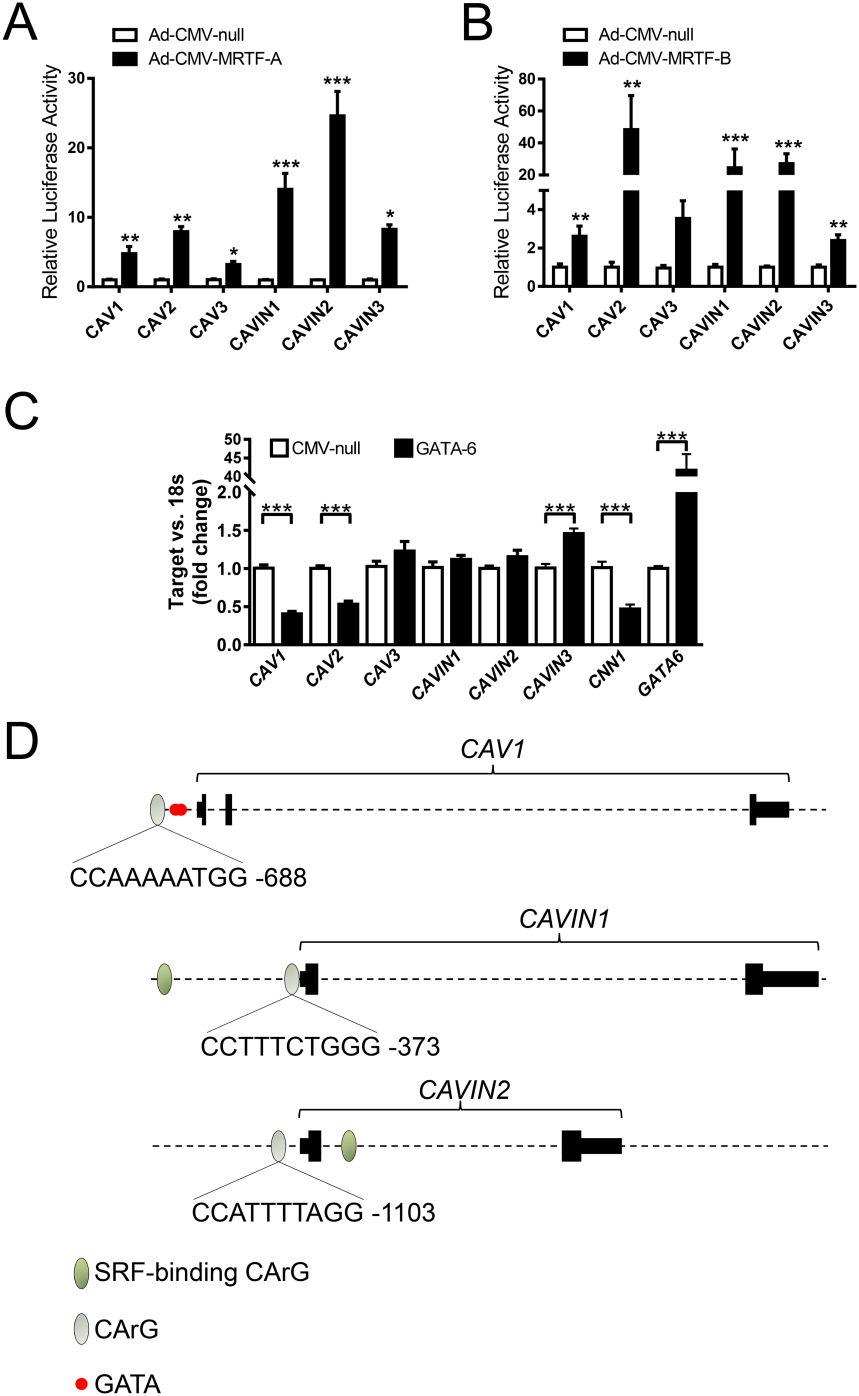
Proximal promoter reporters for caveolins and cavins are activated by MKL1/MRTF-A and MKL2/MRTF-B. Human coronary artery smooth muscle cells were transfected with the indicated promoter reporters and then treated with Ad-CMV-null, Ad-CMV-MRTF-A (A) or Ad-CMV-MRTF-B (B). n = 3–6. In panel C GATA-6 was overexpressed using an adenovirus and the mRNA levels of caveolins and cavins were determined using qRT-PCR. n = 6–12. Panel D shows schematic representations of the CAV1, CAVIN1 and CAVIN2 promoters. MRTFs/SRF binds to DNA via the CC(A/T)_6_GG (CArG) motif and possible (grey) and known (green) SRF-binding CArGs are depicted as ovals. The CAV1 promoter moreover has two GATA motifs depicted in red.

Prior work indicated that two myocardin family coactivators, MYOCD and MKL1/MRTF-A, regulate the mRNA levels of caveolins and cavins in human coronary artery SMCs. MYOCD was also shown to activate a CAV1 promoter reporter [14], but comparable information was not provided for other caveolins and cavins. We therefore first transfected promoter reporter plasmids for CAV1, CAV2, CAV3, CAVIN1, CAVIN2 and CAVIN3 into human coronary artery SMCs, and treated cells with adenoviruses for overexpression of MKL1/MRTF-A and MKL2/MRTF-B, respectively. All promoter reporters were activated by MKL1/MRTF-A and by MKL2/MRTF-B (Fig 1A and 1B), with exception for the CAV3 promoter where the MRTF-B effect failed to reach statistical significance. This argues that proximal promoter sequences are involved throughout. Some qualitative differences were noted; MKL1/MRTF-A was most effective on the CAVIN2 reporter, which was activated 25-fold, whereas MKL2/MRTF-B was most effective on the CAV2 reporter, which was activated 40-fold.

In some instances, such as for telokin, the transcription factor GATA-6 acts by displacement of myocardin family coactivators [19]. Information on how GATA-6 affects caveolins and cavins may thus be valuable for mapping of binding sites in the respective promoter regions. Previous work showed that GATA-6 inhibits CAV1 expression [13], but information on other caveolins and cavins was not available. To examine this, we overexpressed GATA-6 using an adenovirus and determined the effects on caveolin and cavin expression using qRT-PCR. Overexpression of GATA-6 reduced CAV1 as previously reported (Fig 1C). CAV2 was also reduced, but the cavins were unchanged or increased (Fig 1C). We consequently examined the promoter sequence of CAV1. A perfect SRF-binding motif (CArG-box) was found (at -688 bases, Fig 1D) and two GATA-motifs (the one closest to the CArG was at -562 bases, red in Fig 1D). We also searched the CAVIN1 and CAVIN2 promoters for CArG-boxes. A poor match was found in the CAVIN1 promoter whereas a perfect match was found in the CAVIN2 promoter Fig 1D. The latter loci also contain known SRF-binding CArGs (https://genome-euro.ucsc.edu) that are depicted as green ovals.

**Fig 2:**
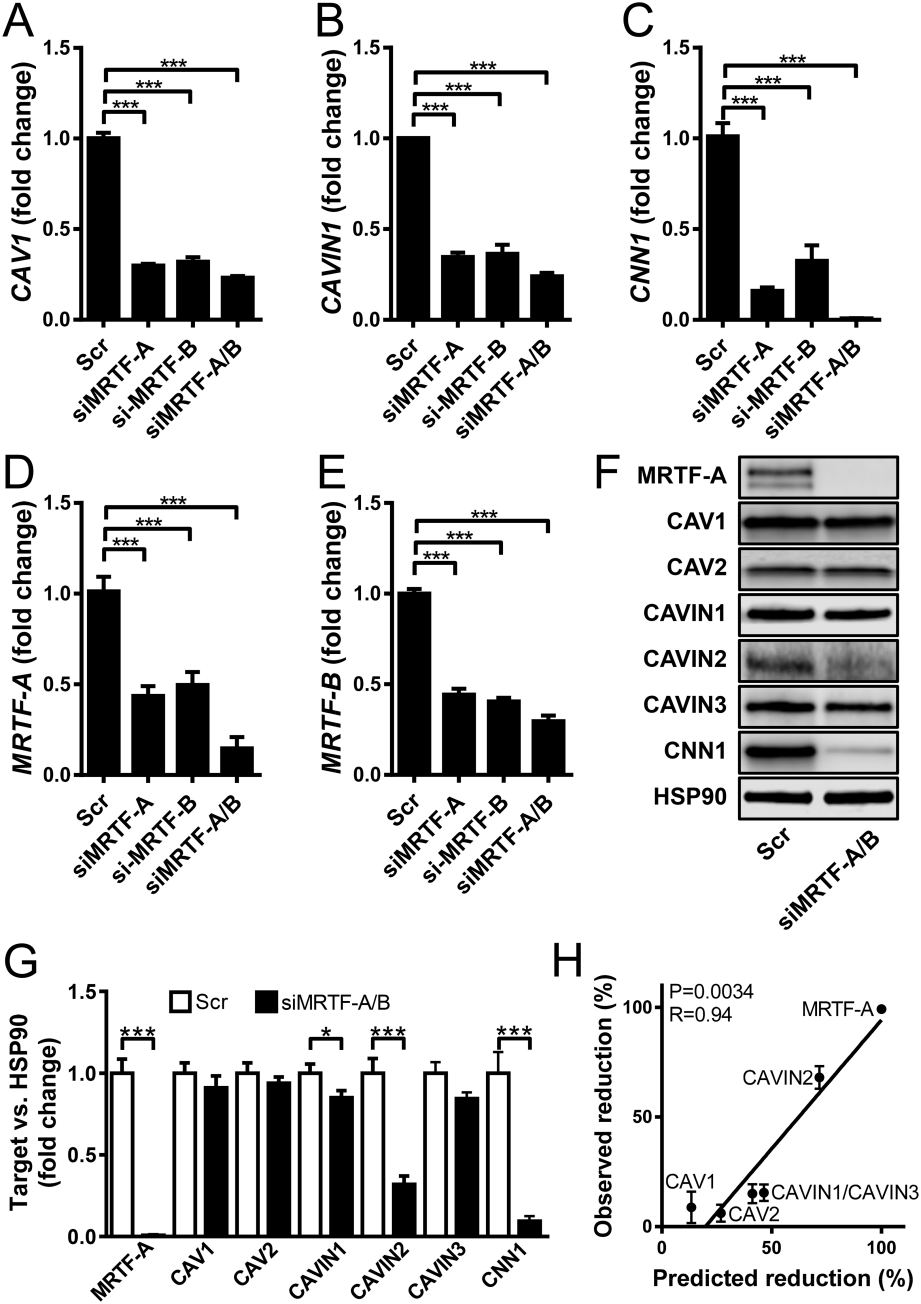
Silencing of MKL1/MRTF-A and MKL2/MRTF-B downregulates CAV1 and CAVIN1. Mouse aortic smooth muscle cells were transfected with scrambled siRNA (Scr) or with siRNAs against MRTF-A, MRTF-B and their combination for 96h. Expression of CAV1, CAVIN1 and the prototypical target gene CNN1 were then analyzed by qRT-PCR (A–C). Knock down was confirmed by qRT-PCR for MRTF-A and MRTF-B (D, E). n = 3–6. Western blotting (F, G) was used to validate MRTF-A repression. n = 12. Among the caveolins and cavins, only CAVIN1 and CAVIN2 were reduced at the protein level. To address if this could be due to slow protein degradation rates, we retrieved half-lives for the caveolins and cavins from a recent study [20] and calculated the expected reduction at 96h assuming exponential decay. The predicted reduction was then plotted versus the observed reduction, revealing a highly significant correlation (Spearman, panel H), and arguing that caveolins are too stable to detect an effect at the protein level in this experimental setting.

We next transfected mouse aortic SMCs with siRNAs against MKL-1/MRTF-A and MKL2/MRTF-B, alone and in combination. Scrambled siRNA was used as control. After 96h cells were harvested and the expression of CAV1 and CAVIN1 was examined by qRT-PCR. In keeping with the promoter reporter results, both CAV1 (Fig 2A) and CAVIN1 (Fig 2B) were reduced by well over 50% following silencing of MKL1/MRTF-A and of MKL2/MRTF-B. The combination of both siRNAs was only marginally more effective. Similar results were obtained for the prototypical target gene calponin (CNN1, Fig 2C). Reduction of MRTF-A and MRTF-B was also examined (Fig 2D and 2E), revealing cross-reactivity of the siRNAs, such that both MRTF-A and MRTF-B were reduced after silencing of MRTF-A, and *vice versa*.

We also examined the effects of MRTF knockdown at the protein level. MRTF-A was convincingly reduced as illustrated in Fig 2F. CAV1, CAV2 and CAVIN3 were unchanged, however, whereas CAVIN1 and CAVIN2 were reduced (Fig 2F and 2G). To examine if the lack of effect on CAV1 and CAV2 could be due to slow protein turnover rates we retrieved protein half-lives for caveolins and cavins from a recent experiment [20] and calculated a predicted reduction at 96h assuming exponential decay. We then plotted the predicted reduction versus the observed reduction and found a tight and highly significant correlation (Fig 2H). From these findings we concluded that the lack of effect on CAV1 and CAV2 proteins in our experiment is due to high protein stability and that longer and stable knockdown is needed to confirm an effect. This may not apply to all experimental conditions as, for example, depolymerization of actin was reported to cause a more rapid decline in CAV1 [14].

**Fig 3:**
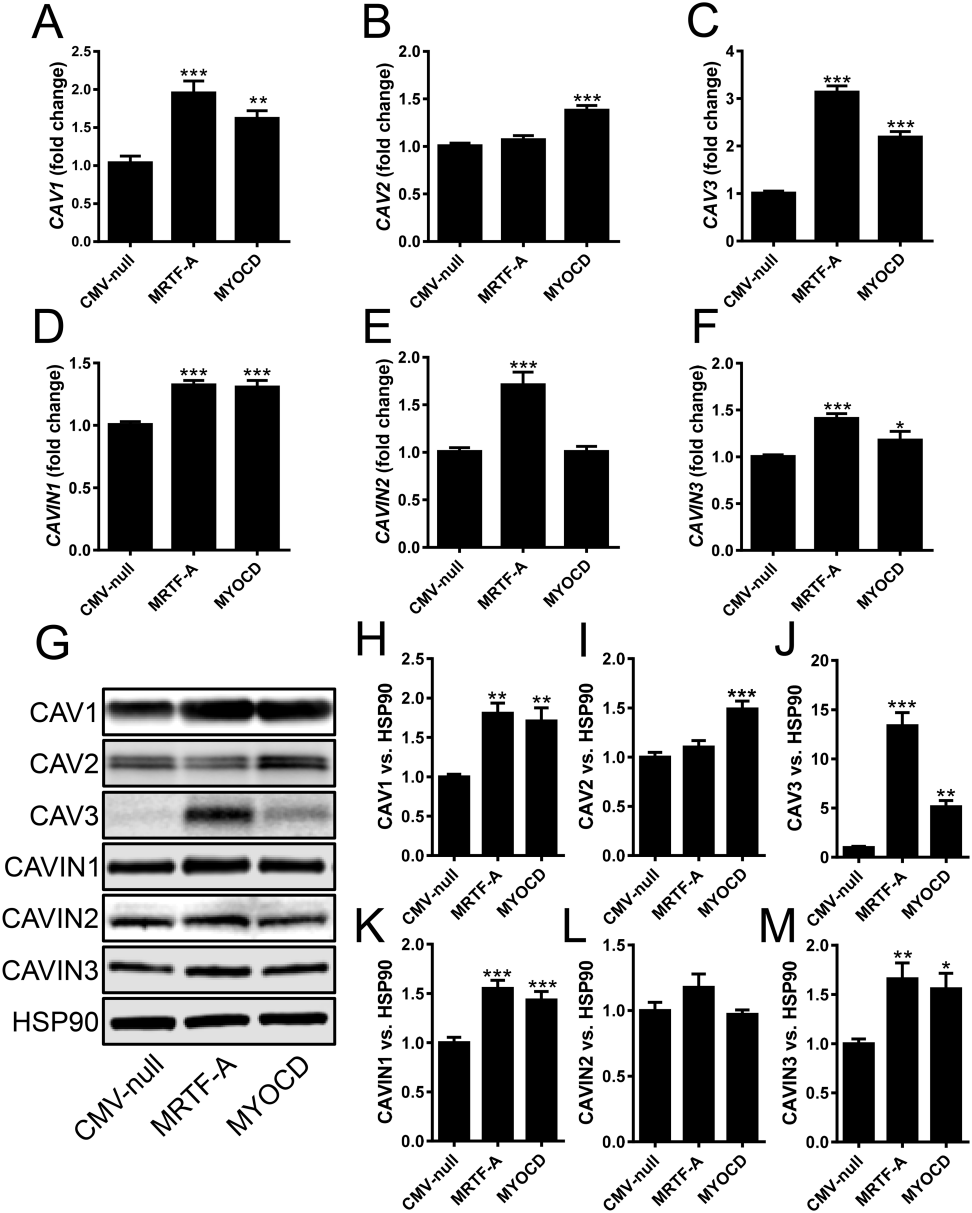
Induction of caveolins and cavins by MKL1/MRTF-A and myocardin in rat bladder smooth muscle. Adenoviral transduction of rat bladder smooth muscle cells with MRTF-A or myocardin (MYOCD) increased mRNA levels of caveolins and cavins (A-F, n = 12–18). Western blotting after transduction demonstrated increases of caveolins and cavins at the protein level that largely matched changes at the mRNA level (G). Summarized data from the western blots is shown in panels H-M (n = 4–12). 18S was used as a house-keeping gene for the qRT-PCR and HSP90 as loading control for western blotting.

Results so far supported a role of myocardin family coactivators in regulation of caveolins and cavins in arterial smooth muscle, but direct evidence that this effect is generalizable was lacking. We therefore examined urinary bladder smooth muscle in which caveolins and cavins have clear-cut functional and morphological roles [21, 22]. First, we isolated rat bladder SMCs and transduced them with adenoviruses for MKL1/MRTF-A and MYOCD. MRTF-A increased the mRNA levels of all caveolins and cavins except CAV2 (Fig 3A through 3F). MYOCD, on the other hand increased all caveolins and cavins except CAVIN2. We next ran western blots to examine whether matching changes occurred at the protein level (Fig 3G; note that protein synthesis rates for caveolins are more rapid than are rates of degradation). Summarized results at the protein level (Fig 3H through 3M) mirrored those at the mRNA level. The only exception was CAVIN2, for which the change with MKL1/MRTF-A did not reach the level of statistical significance (Fig 3L).

**Fig 4:**
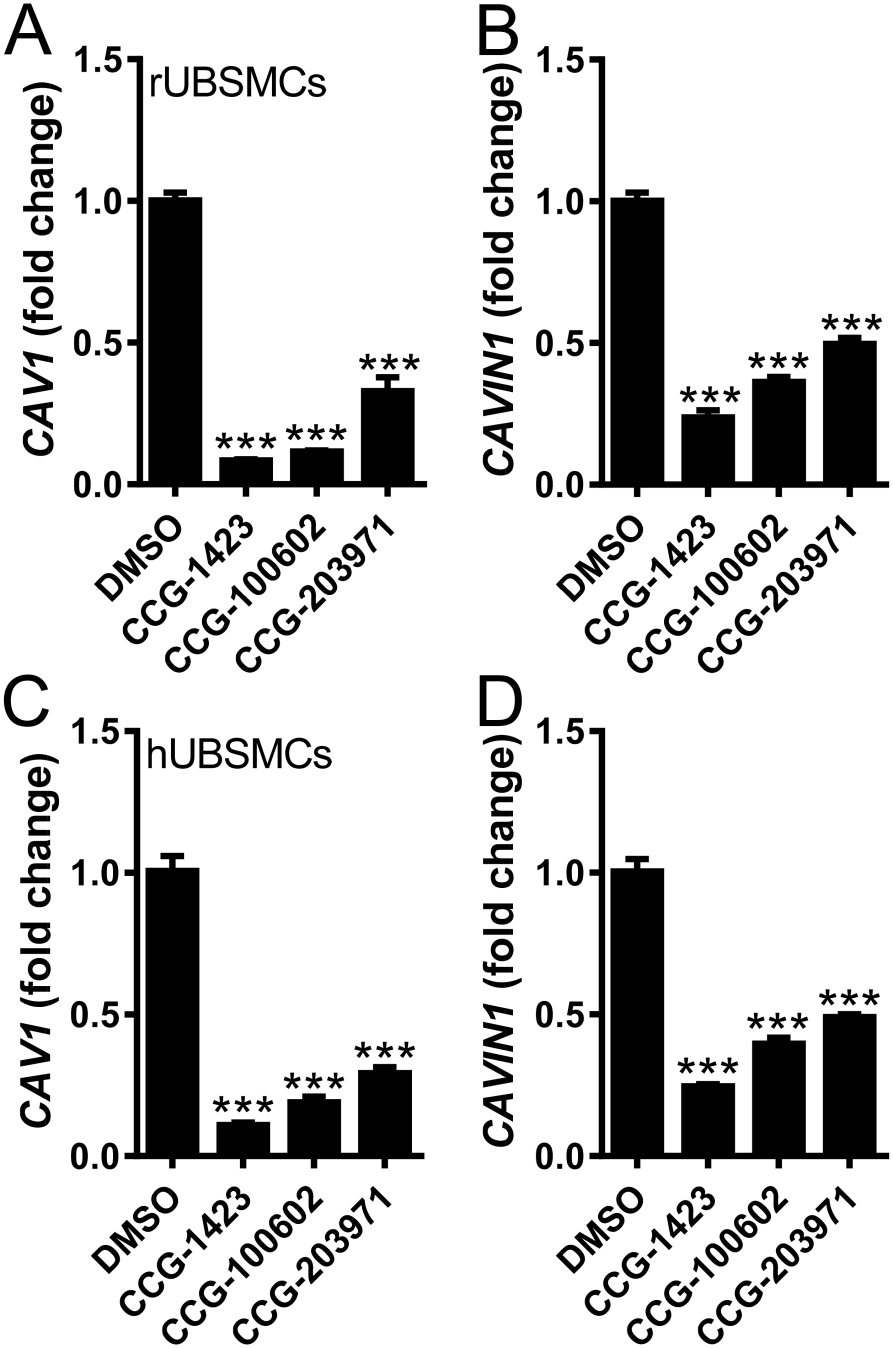
Small molecule inhibitors of MKL signaling reduce CAV1 and CAVIN1 mRNA levels in rat and human bladder smooth muscle cells. Starved rat (A, B) and human (C, D) bladder SMCs were treated with 10 μM of CCG-1423, CCG-100206, CCG203971, or the equivalent volume of DMSO. Expression of caveolin-1 (CAV1, A, C) and cavin-1 (CAVIN1, B, D) was determined by qRT-PCR. 18S was used as a house-keeping gene. n = 5–6.

We next treated rat and human urinary bladder SMCs with first (CCG-1423) and second (CCG-100602, CCG-203971) generation inhibitors of MKL/SRF signaling and examined mRNA levels of CAV1 and CAVIN1. The MKL/SRF inhibitors uniformly reduced expression of CAV1 and CAVIN1 in both rat (Fig 4A and 4B) and human (Fig 4C and 4D) bladder SMCs. CCG-1423 was somewhat more effective in both species and inhibition exceeded 50% in all cases.

**Fig 5:**
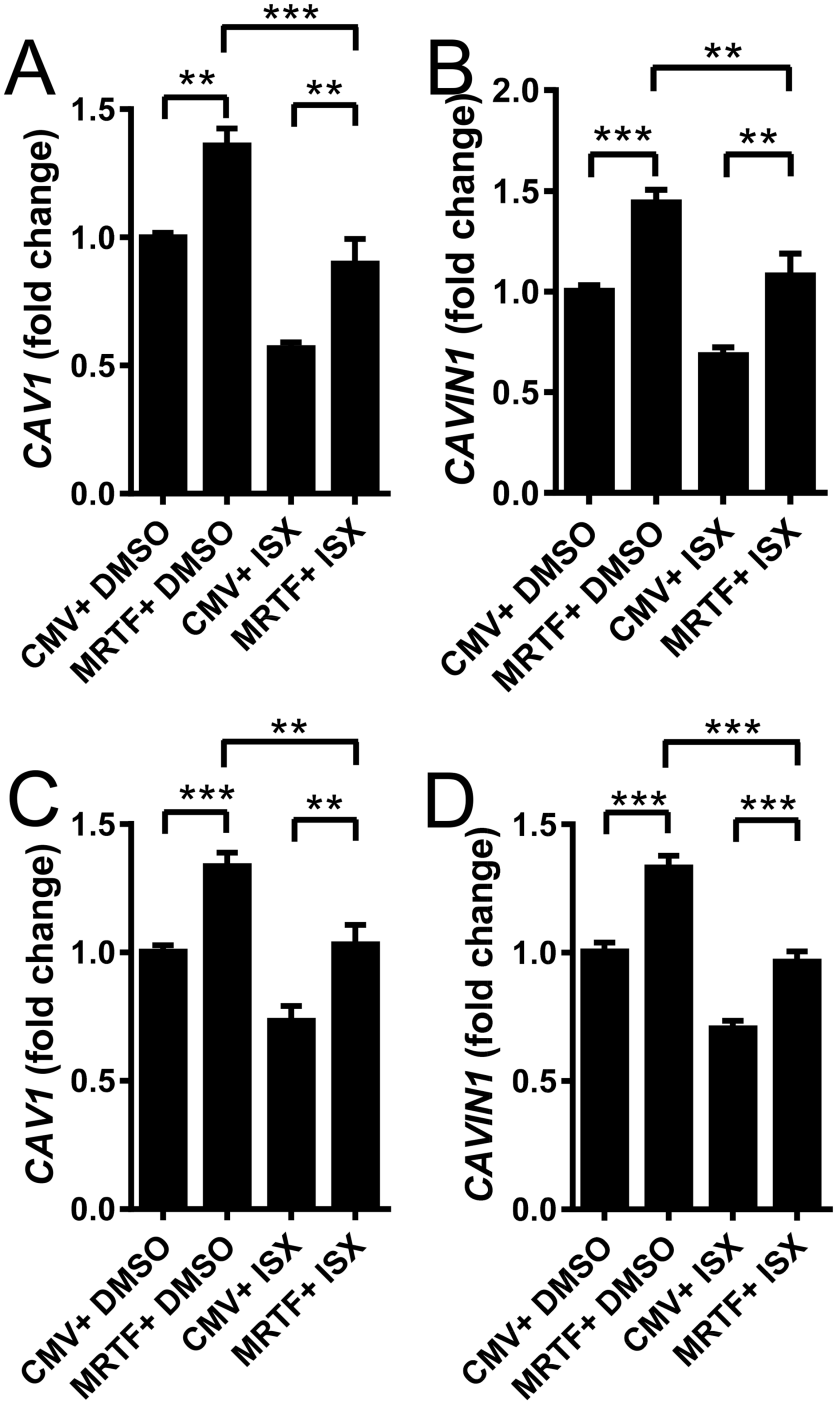
Isoxazole (ISX), an activator of CArG-dependent transcription does not enhance the effect of MRTF-A caveolin-1 or cavin-1. Rat (A, B) and human (C, D) bladder smooth muscle cells were transduced with MKL1/MRTF-A (24h) or control adenovirus and treated with ISX (10μM). Expression of caveolin-1 (CAV1) and cavin-1 (CAVIN1) was determined by qRT-PCR. n = 9–11.

Recent work identified N-cyclopropyl-5-(thiophen-2-yl)-isoxazole-3-carboxamide (ISX) as a small molecule activator of CArG-dependent MKL1/MRTF-A activity [23]. This substance might be of interest to increase CAV1 expression, such as in disease caused by CAV3 mutations. Contrary to our expectation, we were not able to increase the mRNA levels of CAV1 or CAVIN1 in either rat (Fig 5A and 5B) or human (Fig 5C and 5D) bladder SMCs; in fact, ISX significantly repressed MKL1/MRTF-A-stimulated levels.

**Fig 6:**
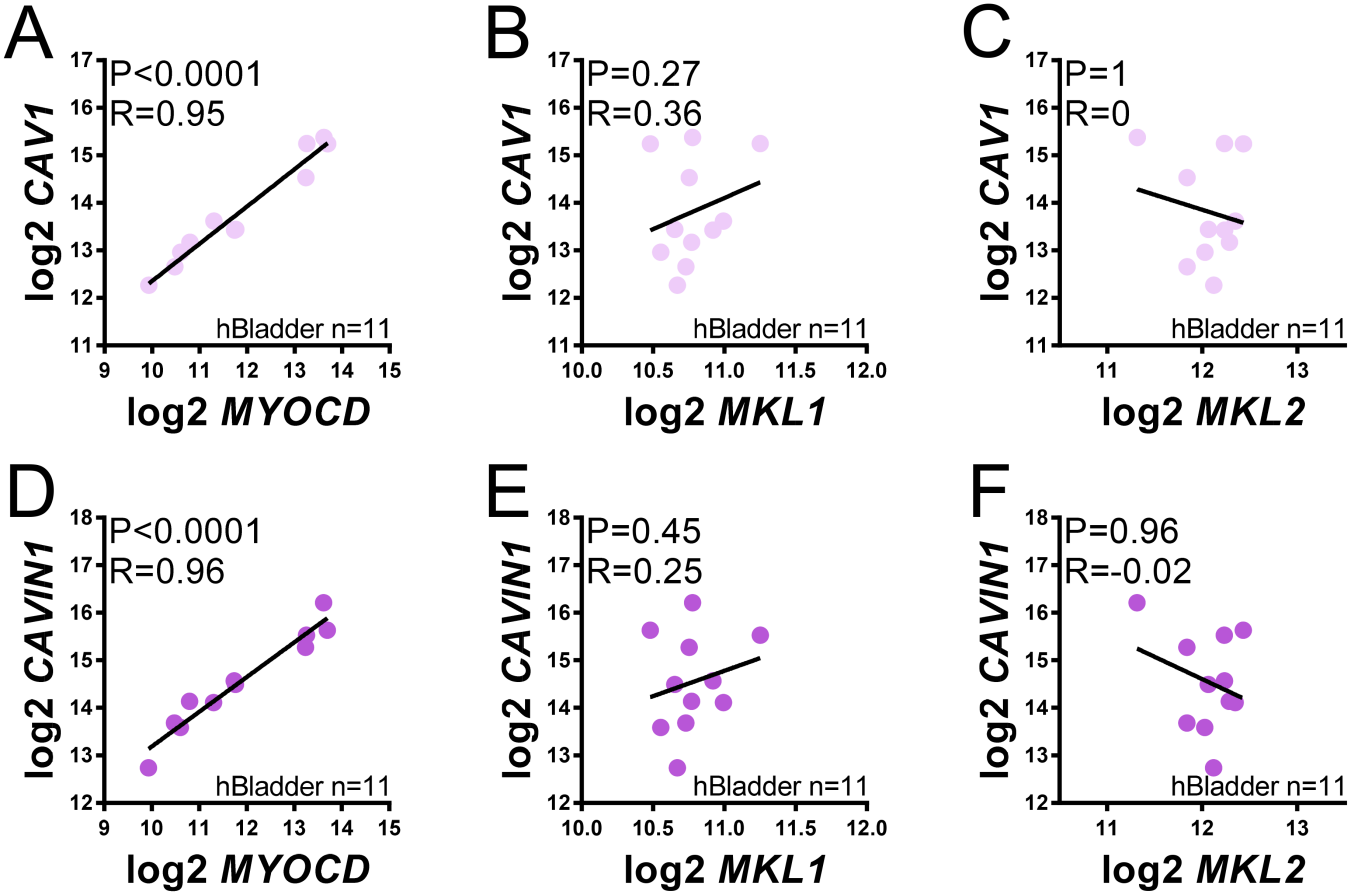
The mRNA level of Myocardin (MYOCD) correlates tightly with the mRNA levels for caveolin-1 (CAV1) and cavin-1 (CAVIN1) in human bladders. Human bladder RNA-Seq data was downloaded from the GTEx Portal and normalized using the trimmed mean of M-values method as described [14]. Correlation coefficients and Spearman Rho coefficients for MYOCD vs. CAV1/CAVIN1 (A, D), MKL1 vs. CAV1/CAVIN1 (B, E) and MKL2 vs. CAV1/CAVIN1 (C, F) are given in the respective panels.

Our inhibitor experiments supported a role for myocardin family coactivators in caveolin and cavin expression in urinary bladder smooth muscle. To examine whether differential expression of CAV1 in human bladders associates with differential expression of myocardin family coactivators we downloaded human bladder RNA-Seq data from the GTExPortal.com. Remarkable correlations between MYOCD and CAV1 and between MYOCD and CAVIN1 were found in this dataset (Fig 6A and 6D). Neither MKL1/MRTF-A nor MKL2/MRTF-B correlated with CAV1 or CAVIN1, suggesting that a major determinant of caveolin and cavin expression in the healthy human urinary bladder is myocardin.

## Discussion

The present study extends previous work showing that myocardin family coactivators control many of the genes responsible for formation of caveolae in human arterial SMCs [14] by showing similar results for rat and human bladder SMCs. We moreover demonstrate that promoter reporters for caveolins and cavins are activated by MKL1/MRTF-A and by MKL2/MRTF-B. The latter finding suggests that approximately 1000 nucleotides in the proximal promoters are enough for regulation of caveolins and cavins. Exact mapping of the DNA-binding regions for MKL1 and MKL2 requires more sophisticated approaches, however. It also seems likely that regions outside of the promoters play a role. The CAV1 promoter, for example, was activated less effectively than the CAVIN2 promoter and yet, in the context of intact chromatin, activation of these genes was similar. Chromatin remodeling may contribute to such discrepancies, but it is also possible that more distal DNA regions play a role.

An inference from previous work is that regulation of CAV1, CAV2 and CAVIN2 depends on the so called SAP domain of the MKLs rather than on the serum response factor (SRF) for which MKLs act as cofactors [4, 14]. SRF binds to well-defined genetic elements called CArG boxes [24], but how the SAP domain acts remains mysterious [4]. Several caveolins and cavins have CArG-boxes in their promoters, but their roles remain to be established. Independent support for an essential role of the SAP domain in regulation of caveolins and CAVIN2 comes from microarrays run for domain deletion mutants of MKL1 [4, 25]. One way to reconcile these observations would be if the SAP domain contributes to binding to some other transcription factor that in turn binds to DNA. We tried here to use GATA-6 to further delineate the mechanism by which MRTFs bind to the caveolin and cavin promoters. While we could confirm the previous finding that CAV1 is repressed by GATA-6 [13], it was apparent that the GATA-6 binding sites were separated from the CArG box by at least 120 nucleotides. This is much farther than the distance between these motifs in the telokin promoter, where GATA-6 is known to displace the MRTFs [19], arguing perhaps that the MRTFs are acting via motifs closer to the GATA motifs. One possibility is that TEADs are involved, and we have noted that there is a perfect MCAT element between the two GATA motifs in the CAV1 promoter. This possibility is currently under investigation.

Here we demonstrate that siRNAs against MKL1 and MKL2 reduce CAV1 and CAVIN1 (cavin-1) mRNA expression by well over 50% in mouse aortic SMCs. Several loss of function approaches, including pharmacological MKL inhibition using CCG inhibitors [26–28], depolymerization of actin, which inhibits MKL activity through cytoplasmic MKL retention [29], and siRNA-driven silencing, thus together support the notion that components of caveolae are under control of myocardin family coactivators (this study and [14]). Whether this control mechanism can be generalized to cell types other than SMCs is not known. In our prior work we presented correlative support for this notion in heart (where MKL2 and CAV1 correlate) and in the esophageal mucosa (where CAV1 and CAVIN1 both correlate with MYOCD) [14], but we note that MKL1 and MKL2 are dramatically repressed during adipogenesis [30] while caveolins and cavins increase [31, 32], arguing that MKL-driven biogenesis of caveolae may not be the rule in all cell types.

ISX is a small molecule MRTF/MKL activator that was recently identified in a screen that used activation of the smooth muscle α-actin promoter reporter as readout [23]. ISX was then shown to promote activity of several CArG-containing reporters and to increase the stability and nuclear localization of MKL1. We were appealed by the possibility to use of ISX for promoting caveolin and cavin expression as this could be of use for therapy of e.g. muscular dystrophy caused by CAV3 mutations, and therefore set out to determine its effect on these genes. We found that both CAV1 and CAVIN1 mRNA levels were reduced by ISX, which was opposite to expectation. A potential explanation is that ISX is acting only on CArG-dependent genes. This may fit with available knowledge on caveolin and cavin regulation via SAP the domain, but other explanations are possible, including specificity towards certain promoters and species differences. We observed similar effects of ISX in rat and human bladder smooth muscle, arguing against species differences as a major explanation.

We found remarkable (Spearman Rho ≥ 0.95) correlations between MYOCD and CAV1, and between MYOCD and CAVIN1, in the human urinary bladder. These observations further support the idea that myocardin family coactivators are important determinants of caveolin and cavin expression in human tissues. Some aspects of these analyses are enigmatic, however. For instance, MKL inhibitors had a sizeable effect on CAV1 and CAVIN1 expression in human bladder SMCs, and yet neither MKL1 nor MKL2 correlated with CAV1/CAVIN1 in intact bladder tissue. A potential explanation is that the MKLs assume a more prominent role in subcultures of SMCs where, in fact, the MYOCD mRNA level is known to drop compared to intact tissue [33]. Another, equally plausible, explanation is that MYOCD is constitutively nuclear whereas MKL1 and MKL2 translocate between the cytoplasm and nucleus depending on cellular activation state [29, 34]. As a consequence, the mRNA level of MYOCD is expected to be a better measure of its transcriptional impact than are the mRNA levels of MKL1 and MKL2, where instead the nucleocytoplasmic distribution is the activation determinant [29].

SMC-specific deletion of myocardin in adult mice causes a number of urogenital phenotypes [35] as does deletion of either caveolin-1 [21, 36, 37] or cavin-1 [22]. Close phenotype comparisons suggest that the phenotypes have little in common, however. In cavin-1/caveolin-1 knockout animals there is bladder hypertrophy occurring preferentially in males, but no apparent difference in the relative thickness of the muscle layer, and yet depolarisation-induced contraction is reduced [22, 36]. In the inducible myocardin knockouts a marked dilation of the bladder with drastic thinning of the muscle cell layer and apparent detachment of the mucosal layer is seen [35]. Such differences are to be expected, because myocardin targets a host of genes that are critical for contractility and survival, but it cannot be ruled out that loss of caveolae contributes in some way to the phenotype of myocardin knockouts. It would indeed be of considerable interest to examine the membrane density of caveolae in inducible myocardin knockout animals.

To summarize, the present study demonstrates that the myocardin family coactivators MKL1 and MKL2 regulate most caveolins and cavins via proximal promoter sequences, that silencing of MKL1 and MKL2 via RNA interference reduces expression of caveolin-1 and cavin-1 and that MKL/MYOCD-dependent control of caveolins and cavins can be generalized to include smooth muscle in the urogenital system. Myocardin family coactivators therefore emerge as important, if not major, determinants of caveolae formation in smooth muscle.

## Supporting information

S1 File Data for Fig 1D. (PDF)

S1 Table Data for Fig 1A. (PDF)

S2 Table Data for Fig 1B. (PDF)

S3 Table Data for Fig 1C. (PDF)

S4 Table Data for Fig 2A through 2E. (PDF)

S5 Table Data for Fig 2G. (PDF)

S6 Table Data for Fig 2H. (PDF)

S7 Table Data for Fig 3A through 3F. (PDF)

S8 Table Data for Fig 3H through 3M. (PDF)

S9 Table Data for Fig 4A and 4B. (PDF)

S10 Table Data for Fig 4C and 4D. (PDF)

S11 Table Data for Fig 5A and 5B. (PDF)

S12 Table Data for Fig 5C and 5D. (PDF)

S13 Table Data for Fig 6A through 6F. (PDF)
