# Supplementary material for: Similar regulatory mechanisms of caveolins and cavins by myocardin family coactivators in arterial and bladder smooth muscle
Source: PLoS One. 2017 May 25;12(5):e0176759. doi: 10.1371/journal.pone.0176759 (PMC5444588; doi:10.1371/journal.pone.0176759)
Supplement: S1 File — (PDF) [file pone.0176759.s001.pdf]

S1 File. Data for Fig1 D

CAV1 promoter sequence:

GGAGGTGTTATTTACCCGAGTCCTGGGGACAGTCCCCGGGACTCTCCGCCAGGCGCCCAGACCGGCAGGTCCCGCAGGCGGCGCGCGGTGTGT
TTGCACTTTCCAAAGTTCTTGAACCATCTCAAGAACTCCTTCTGCATCTTGGCGTCTGGCAGGGGTGTTCCGAGAGAGGTAGACCTCCCCTCCCCA
AACTGCCACCATCACTTCCAACGCCCTCCACGCGCTGGAGCTCTGCCCGGGTGTGGAAACCTCGTCTTCCAACACGTAGCTGCCCTTCAGCCACC
CGCCCGCAGCCTGGGAGTGCCCTGAGGGTGGGTCGGGGGAGCTGCGCAGGTGAGACTGAGTTCTAGGACATTTAGGGGGTCTGGTGCCTGGCT
CCG**CCAAAAATGG**GGACTTTCGGGATTGTGATCATCACGGCGGATTGAGCAGGGAGAGCCGTGGAGGGACAAGAGAGGGCCGAGGCAGGGTGG
GGGGCGCGGGCAGGTGCGAGGGGGATGCGGCCAAGAAGCAGCGATAAAGGGAA**CATTCC**ACGGGTCGGGCGGCTGCTGTTGGATCTTAGATAA
AGCTGGAAGGGATTACCGGGGCAGGGGTAATAGGGACCGGGGACGGGAACGCGAAACAGGTGAAGCGCTCAGGGCGAGAGCGACTCGGCTTAG
GGAGTCCGGGAGAAGCCTGCGGCTGCCCCCTCGCCGCCGAGGTCCTGCGGGTCCTGCGGGTCCTGCGTGCTGAGCCGGGGCGTGCGCGGGCG
GGGGCCTTCGGACCGCGCGGCGGGGCCTGCCCTGACCCCTGGCGGCGGGCGGGGGAGGCAGGCGCGCCCTGCAGAGTACAGAGGGGTGTGG
TGTCCTCTGCGAGATCCTCTTAAAAAGCTGGCTACGCGCAGGCGGTTTCTGTGCACGGAGCCGTAGCTGTCGGAGCGGTTAGTTCGATTTCGAGC
TCGAGGTTTCCCCCGCCGCCAGGCTGACTTCTCATCGCTTGTTTTTCTTTTTGCATTTTTCCTCCCACCGCCGTTGCCGCCCTCCCCGTCCTGGCC
GTCCGCCCTCCGCCCTCTGCAGGGACATCTCTACACCGTTCCCATCC

Summary: Perfect CArG: -688 to -697; Two GATA seq; One TEAD motif

CAVIN1 promoter sequence:

CGTTTGATTGCCCATCTTAATTCTACTCTGTTAGGGCAATTCTAAAAAGAACTTAGAGGAGCTTCGCAGCCCTTCACCACCACCGTGACCCCAAATACG
TTCTCTCTTTCTCCCCCCGCCCCCCACAAACAACCCCCTAAGCTTACCCCTGAGTTAGTAACCTGGTTGGGGTTGGTGATGAGGAAATCCGAGAGGCCT
CTTAGAAAATATTGTAAAGGGCAAGGGTCCTTTTGTGCTCAAAATCTAGGTAAAGGCAAATACAAGATTTTGCCTATAATTTCAGAATCTCCTGGTCC
ACCCGGCTCCCAGATCTTGAGGATTTTCGGATGAGCAATTCAAGTGAGAGGGAAACAGAGAGCAACACTCTTAGAACCTGATTCCGTTCCTCAGCTCG
CATTCCTCCGCCCGTCCCGCTCCCGCCGCCAGCGGAGGCCCTAGTCTCCCGCTCCAACTATTCCAACCATCCCGGGAAGGGTGGGGCGCTCGGCTCTTG
GGTCCCCCTCCGCCGCCCCGCCTCGTCGATCTCCCCTTCTGCCCCGGTCCCTC**CCTTTCTGGG**GTGGGGCCAGCCAATCAGCGATCAGACTCCGGAGT
TTGGCCCGGGAGCTGGGGAGCTCACCGATCCCCCGCCCAGCAGTTCTGGCCGCTGTCCCGGTGCGCACGGACGTGGCTCGAGTTTCCTCTGCTCTCCGC
TCTCGCCCGCTAGCTCTCCTCCCTTCCGCTCCTGCTTCTCTCCGGGTCTCCCGCTCCAGCTCCAGCCCCACCCGGCCGGTCCCGCACGGCTCCGGGTAGC
CATGGAGGACCCCACGCTCTATATTGTCGAGCGGCCGCTTCCCGGGTACCCCGACGCCGAGGCCCCGGAGCCTTCCTCCGCTGGGGCTCAGGCAGCGG
AGGAGCCGTCGGGGGCCGGCTCAGAAGAGCTGATCAAGTC

Summary: Poor (-382 to -373): CCTTTCTGGG

CAVIN2 promoter sequence

ACGAATGGCATTGTTCCATTATCAATCCCTGACCTGGTATGAGCTGGCATTTGTTCGTCTCAAAATTGTTCATAGAAGGCCTATTACAAACTTCCTGCT
GAAAGCACTTTCAAAGGCTGGATACGTTTAAAATCAACTCAAGCATGTAAAG**CCATTTTAGG**AAAATAAGCCCCACATCCCAAGGTTCAGCTGAGGA
GGGCCACATTTGAGAACAGGTGTGCCACTTCACTGAGCAGGAATTTTAAAATGTCAAACATTAGAACCACTTGAGAAACTCCAACTCAGCCTGCCAAC
AGACACTTCTGGGCACGTGTCAATAAATGCCTTGCAGAGATGGGCATTTGAGAAAGTCTCTCCTCACCAACTCAAAGTTAGTTAAAACAAATCATCAC
GGTAGGTTCTTAAAAAGACCACATTTTATTTTTCCTCAGTCTCACTTCTTGAAAAAAACAATCATTTTTTTAAATGTGGTCATTCAAAAAGTTTCCATAT
ATTACATAACAGTCATAACAGCAGTATTACTTTTTTTAAATATCCCTGAAGTTCAATCTCTTTTAACTTATTTTGTATCTGCAACTGCAAGAGTAATAAG
AACTGAGCTGTTTTCCTTATCATTATCAGAGTTAGTCCCATTCCTTTGCAAATGAGAAACTAATAATACAAACTTGGCTGCAGTACCCTTTAAACTCTA
TGCCTCCCAGTAGGACACCCGGTGTTCTGCAACCCATCAGTCAGTTCTAGGGCAAATGCAAACAATACTTTCCGTTACAATAACTTGTATCCAGGAAA
AAGAAACAGGCACCCTGTTGCATCTGCAGCATAGCAGCCAGAGAAAAGTTTACCAGCTTTAGCCAATTGGAAGCTGTGGAGCTGGGCCAAGTTAGCA
TGTTTTTCTCCGCCTCTAGATGAAGTCATGATTGAAAT***ATG***ACATTGAAAGGGAAGGGGAACAGGAAAGAAAAGCTAAACCTGCAACAAAGTGTCCT
GTGCCCAGAACGCGGTTAGGAAGTGTGTGCATACGTCTGAACCCTAA***ATG***GTTCTCAGTTCTGTAAACTTCTCCTCCCACTGGGTGGAGTAGGGCCTTT
AAGAGCAGCTGGA***ATG***CAGTTCCCCTGATCAGCGTAGCCAGTTGTTGCCTGTCTGAACCTCTGCCAGTCCTGGAGACTGGTGCCCTGAGCTCCAACCA
GCGGGCCTCATCCTACACCCTCACCACCGCAACTTCTCACCCGAGCAAGAAGCAGCTCCCAGAGAGAAAGAACGTTCCCACCT

Summary: Poor (CCCTAAATGG): -239 to -230; Good (CCATTTTAGG): -1112 to -1103
