# Supplementary material for: Similar regulatory mechanisms of caveolins and cavins by myocardin family coactivators in arterial and bladder smooth muscle
Source: PLoS One. 2017 May 25;12(5):e0176759. doi: 10.1371/journal.pone.0176759 (PMC5444588; doi:10.1371/journal.pone.0176759)
Supplement: S1 Table — (PDF) [file pone.0176759.s002.pdf]

S1 Table Data for Fig1 A

	Control (fold change)						MKL1 (fold change)					
CAV1	0.82	0.77	1.41	0.92	0.83	1.25	2.92	2.64	2.21	8.41	6.16	6.34
CAV2	0.92	0.83	1.25				9.37	7.12	7.30			
CAV3	0.83	1.17	0.88	1.29			3.39	5.19	2.96	3.11	2.21	2.48
CAVIN1	1.25	0.91	0.84	0.99	1.01		8.80	8.20	12.70	22.37	13.14	19.03
CAVIN2	0.99	1.01	1.11	0.93	0.95		23.77	14.54	20.43	34.79	29.48	
CAVIN3	1.24	0.76	1.19	0.81			9.64	7.48	6.57	9.99	7.72	
