# Supplementary material for: Similar regulatory mechanisms of caveolins and cavins by myocardin family coactivators in arterial and bladder smooth muscle
Source: PLoS One. 2017 May 25;12(5):e0176759. doi: 10.1371/journal.pone.0176759 (PMC5444588; doi:10.1371/journal.pone.0176759)
Supplement: S3 Table — (PDF) [file pone.0176759.s004.pdf]

S3 Table Data for Fig1 C

Targets		$2^{-\Delta\Delta CT}$ (18S as HK gene)											
CAV1	CMV-null	1.08	1.15	0.96	0.91	1.04	0.88						
	CMV-GATA-6	0.45	0.52	0.39	0.44	0.35	0.29						
CAV2	CMV-null	1.01	1.08	1.06	1.02	1.01	0.85						
	CMV-GATA-6	0.64	0.63	0.54	0.55	0.42	0.42						
CAV3	CMV-null	0.74	1.1	1.1	0.67	1.26	1.32	0.68	0.96	1.03	1.19	0.92	1.34
	CMV-GATA-6	1.89	1.17	1.51	0.96	0.56	1.13	1.67	0.9	1.44	1.05		
CAVIN1	CMV-null	0.87	1.02	0.96	0.81	1.13	1.3						
	CMV-GATA-6	0.9	1.19	1.05	1.1	1.26	1.2						
CAVIN2	CMV-null	1.01	1.12	1.06	0.94	0.95	0.94						
	CMV-GATA-6	1.37	1.44	1.12	1	1.1	0.91						
CAVIN3	CMV-null	0.97	1.25	0.95	1.01	0.97	0.88						
	CMV-GATA-6	1.47	1.64	1.61	1.48	1.29	1.25						
CNN1	CMV-null	0.91	1.38	0.94	1.03	0.92	0.89						
	CMV-GATA-6	0.52	0.69	0.54	0.42	0.37	0.27						
GATA-6	CMV-null	1.04	1.12	1.01	0.92	0.95	0.98						
	CMV-GATA-6	44.3	53	46.7	48	33.7	24.4						
