# Supplementary material for: Similar regulatory mechanisms of caveolins and cavins by myocardin family coactivators in arterial and bladder smooth muscle
Source: PLoS One. 2017 May 25;12(5):e0176759. doi: 10.1371/journal.pone.0176759 (PMC5444588; doi:10.1371/journal.pone.0176759)
Supplement: S4 Table — (PDF) [file pone.0176759.s005.pdf]

S4 Table Data for Fig2 A to E

Targets	$2^{-\Delta\Delta CT}$ (18S as HK gene)						
CAV1 (Panel A)	Scr	1.012	0.992	0.996	0.879	1.098	1.036
	siMRTF-A	0.301	0.324	0.284	0.283	0.330	0.267
	si-MRTF-B	0.357	0.380	0.249	0.334	0.360	0.243
	siMRTF-A/B	0.265	0.229	0.237	0.232	0.237	0.194
CAVIN1 (Panel B)	Scr	1.050	1.020	0.934	1.085	0.992	0.929
	siMRTF-A	0.262	0.328	0.385	0.428	0.321	0.357
	si-MRTF-B	0.391	0.536	0.228	0.362	0.444	0.223
	siMRTF-A/B	0.276	0.210	0.275	0.278	0.160	0.238
CNN1 (Panel C)	Scr	0.979	1.137	0.898	1.302	0.944	0.813
	siMRTF-A	0.114	0.139	0.217	0.203	0.113	0.175
	si-MRTF-B	0.200	0.231	0.633	0.155	0.189	0.548
	siMRTF-A/B	0.012	0.004	0.012	0.009	0.002	0.009
MRTF-A (Panel D)	Scr	1.134	0.882	0.766	1.139	1.146	
	siMRTF-A	0.298	0.461	0.339	0.602	0.483	
	si-MRTF-B	0.547	0.674	0.257	0.584	0.420	
	siMRTF-A/B	0.032	0.044	0.064	0.316	0.281	
MRTF-B (Panel E)	Scr	1.043		0.957		1.002	
	siMRTF-A	0.411		0.413		0.507	
	si-MRTF-B	0.433		0.366		0.416	
	siMRTF-A/B	0.238		0.301		0.348	
