# Supplementary material for: Similar regulatory mechanisms of caveolins and cavins by myocardin family coactivators in arterial and bladder smooth muscle
Source: PLoS One. 2017 May 25;12(5):e0176759. doi: 10.1371/journal.pone.0176759 (PMC5444588; doi:10.1371/journal.pone.0176759)
Supplement: S5 Table — (PDF) [file pone.0176759.s006.pdf]

S5 Table Data for Fig2 G

Targets		Targets over HSP90											
MRTF-A	Scr	1.03	1.12	0.49	1.18	1.19	1.00	1.06	1.31	1.50	0.78	0.55	0.80
	siMRTF-A/B	0.01	0.02	0.01	0.02	0.01	0.01	0.00	0.00	0.00	0.01	0.00	0.00
CAV1	Scr	0.78	0.86	0.96	0.84	1.44	1.12	1.07	0.79	0.99	1.21	0.71	1.23
	siMRTF-A/B	0.73	0.82	0.61	1.36	0.75	1.38	0.65	0.81	0.93	0.89	0.94	1.06
CAV2	Scr	1.36	0.62	0.95	1.36	0.78	0.93	0.95	0.80	1.10	1.13	1.02	1.01
	siMRTF-A/B	0.79	1.05	1.09	0.96	0.79	1.12	0.88	0.74	0.96	0.95	0.84	1.10
CAVIN1	Scr	0.65	0.86	1.21	1.03	1.21	1.05	0.93	0.75	0.98	0.92	1.16	1.27
	siMRTF-A/B	0.66	0.95	0.63	0.93	0.87	0.90	0.65	0.74	0.89	0.85	1.02	1.10
CAVIN2	Scr	0.76	0.63	0.77	1.22	1.09	1.52	1.05	0.77	0.79	0.84	1.61	0.94
	siMRTF-A/B	0.21	0.15	0.33	0.20	0.58	0.74	0.44	0.23	0.25	0.24	0.19	0.27
CAVIN3	Scr	1.53	0.98	0.74	0.83	0.87	1.05	1.36	0.91	0.84	1.04	1.02	0.82
	siMRTF-A/B	0.99	0.66	0.73	0.64	0.83	0.89	1.00	0.96	0.89	0.70	0.91	0.94
CNN1	Scr	1.33	0.60	1.19	1.00	0.90	0.99	1.19	0.34	0.56	0.80	2.09	1.01
	siMRTF-A/B	0.03	0.02	0.04	0.01	0.02	0.03	0.07	0.06	0.09	0.27	0.33	0.16
