# Supplementary material for: Similar regulatory mechanisms of caveolins and cavins by myocardin family coactivators in arterial and bladder smooth muscle
Source: PLoS One. 2017 May 25;12(5):e0176759. doi: 10.1371/journal.pone.0176759 (PMC5444588; doi:10.1371/journal.pone.0176759)
Supplement: S6 Table — (PDF) [file pone.0176759.s007.pdf]

## S6 Table Data for Fig2 H

| | Predicted reduction (%) | Observed reduction (%) | |
|---|---|---|---|
| | | Mean | SEM |
| CAV1 | 13.40 | 8.87 | 7.19 |
| CAV2 | 26.88 | 6.18 | 3.82 |
| CAVIN1 | 41.25 | 15.05 | 4.35 |
| CAVIN2 | 71.69 | 68.07 | 5.16 |
| CAVIN3 | 46.44 | 15.49 | 3.78 |
| MRTF-A | 99.99 | 99.11 | 0.19 |
