# Supplementary material for: Similar regulatory mechanisms of caveolins and cavins by myocardin family coactivators in arterial and bladder smooth muscle
Source: PLoS One. 2017 May 25;12(5):e0176759. doi: 10.1371/journal.pone.0176759 (PMC5444588; doi:10.1371/journal.pone.0176759)
Supplement: S8 Table — (PDF) [file pone.0176759.s009.pdf]

S8 Table Data for Fig3 H to M

Targets	Targets over HSP90												
CAV1 (Panel H)	CMV-null	1.08	0.98	0.92	1.02								
	MRTF-A	2.15	1.52	1.80	1.76								
	MYOCD	1.63	1.27	2.00	1.93								
CAV2 (Panel I)	CMV-null	0.81	0.92	0.99	1.28	1.14	1.15	1.07	0.65	0.97	0.91	1.08	1.04
	MRTF-A	0.89	0.89	0.77	1.25	1.44	1.37	1.11	0.80	1.04	1.29	1.24	1.15
	MYOCD	1.22	1.50	1.35	1.72	1.92	1.20	1.86	1.04	1.53	1.71	1.26	1.56
CAV3 (Panel J)	CMV-null	0.67	1.25	0.81	1.26	1.20	0.65	1.65	0.50	1.02	1.08	1.49	0.41
	MRTF-A	12.36	18.14	15.36	22.11	15.78	13.62	17.40	10.90	9.66	10.43	8.78	5.92
	MYOCD	4.42	6.39	6.74	8.22	8.16	6.54	5.59	1.58	3.79	5.17	3.08	1.97
CAVIN1 (Panel K)	CMV-null	0.91	1.12	0.91	1.06	0.83	1.18	1.29	0.70	0.94	0.78	1.28	1.00
	MRTF-A	1.39	1.50	1.18	1.53	1.34	1.90	2.01	1.21	1.28	1.65	1.91	1.72
	MYOCD	1.21	1.48	1.37	1.35	1.71	1.84	1.27	1.56	0.71	1.48	1.57	1.65
CAVIN2 (Panel L)	CMV-null	0.91	1.04	0.99	1.06	1.05	1.33	0.93	0.70				
	MRTF-A	1.26	1.16	0.87	1.46	1.54	1.37	1.05	0.72				
	MYOCD	0.90	1.00	0.98	1.07	1.08	1.04	0.89	0.81				
CAVIN3 (Panel M)	CMV-null	1.11	0.99	1.04	0.85	0.99	0.94	1.25	0.82				
	MRTF-A	1.47	1.17	1.29	1.24	1.93	1.94	2.49	1.76				
	MYOCD	1.40	1.00	1.16	1.10	2.05	2.05	1.94	1.77				
