# Supplementary material for: Similar regulatory mechanisms of caveolins and cavins by myocardin family coactivators in arterial and bladder smooth muscle
Source: PLoS One. 2017 May 25;12(5):e0176759. doi: 10.1371/journal.pone.0176759 (PMC5444588; doi:10.1371/journal.pone.0176759)
Supplement: S9 Table — (PDF) [file pone.0176759.s010.pdf]

S9 Table Data for Fig4 A and B

Targets	2-ΔΔCT (18S as HK gene)						
CAV1 (Panel A)	DMSO	0.887	1.037	1.086	0.975	0.992	1.034
	CCG-1423	0.075	0.072	0.076	0.091	0.097	0.096
	CCG-100602	0.118	0.117	0.104	0.119	0.129	0.106
	CCG-203971	0.241	0.207	0.209	0.454	0.432	0.431
CAVIN1 (Panel B)	DMSO	0.980	1.060	0.962	0.894	1.070	1.046
	CCG-1423	0.171	0.184	0.184	0.281	0.293	0.308
	CCG-100602	0.424	0.353	0.356	0.381	0.374	0.291
	CCG-203971	0.428	0.480	0.453	0.520	0.527	0.573
