# Supplementary material for: Similar regulatory mechanisms of caveolins and cavins by myocardin family coactivators in arterial and bladder smooth muscle
Source: PLoS One. 2017 May 25;12(5):e0176759. doi: 10.1371/journal.pone.0176759 (PMC5444588; doi:10.1371/journal.pone.0176759)
Supplement: S10 Table — (PDF) [file pone.0176759.s011.pdf]

S10 Table Data for Fig4 C and D

Targets	2-ΔΔCT (18S as HK gene)						
CAV1 (Panel C)	DMSO	1.242	0.882	0.912	0.964	0.988	1.050
	CCG-1423	0.142	0.116	0.096	0.094	0.097	
	CCG-100602	0.130	0.166	0.231	0.224	0.200	
	CCG-203971	0.362	0.306	0.304	0.245	0.246	
CAVIN1 (Panel D)	DMSO	0.910	1.141	0.963	0.882	1.013	1.120
	CCG-1423	0.237	0.231	0.240	0.251	0.274	
	CCG-100602	0.344	0.374	0.405	0.397	0.466	
	CCG-203971	0.502	0.470	0.528	0.471	0.481	
