# Supplementary material for: Similar regulatory mechanisms of caveolins and cavins by myocardin family coactivators in arterial and bladder smooth muscle
Source: PLoS One. 2017 May 25;12(5):e0176759. doi: 10.1371/journal.pone.0176759 (PMC5444588; doi:10.1371/journal.pone.0176759)
Supplement: S11 Table — (PDF) [file pone.0176759.s012.pdf]

S11 Table Data for Fig5 A and B

Targets	2-ΔΔCT (18S as HK gene)									
CAV1 (Panel A)	CMV+ DMSO	1.016	0.965	1.021	0.923	1.019	1.063	0.936	1.056	1.012
	MRTF+ DMSO	1.366	1.293	1.241	1.137	1.167	1.229	1.699	1.603	1.496
	CMV+ ISX	0.648	0.560	0.706	0.522	0.576	0.509	0.523	0.524	0.549
	MRTF+ ISX	1.246	1.168	1.180	0.562	0.503	0.573	0.957	0.994	0.902
CAVIN1 (Panel B)	CMV+ DMSO	0.890	1.085	1.036	0.945	1.067	0.992	0.892	1.132	0.991
	MRTF+ DMSO	1.624	1.627	1.719	1.170	1.277	1.285	1.537	1.405	1.348
	CMV+ ISX	0.760	0.829	0.823	0.574	0.664	0.485	0.656	0.692	0.681
	MRTF+ ISX	1.351	1.426	1.330	0.685	0.587	0.694	1.261	1.220	1.166
