# Supplementary material for: Similar regulatory mechanisms of caveolins and cavins by myocardin family coactivators in arterial and bladder smooth muscle
Source: PLoS One. 2017 May 25;12(5):e0176759. doi: 10.1371/journal.pone.0176759 (PMC5444588; doi:10.1371/journal.pone.0176759)
Supplement: S12 Table — (PDF) [file pone.0176759.s013.pdf]

S12 Table Data for Fig5 C and D

Targets		2-ΔΔCT (18S as HK gene)										
CAV1 (Panel C)	CMV+ DMSO	1.04	1.15	0.84	0.98	0.99	1.03	1.07	0.93	0.98	0.95	1.07
	MRTF+ DMSO	1.48	1.40	1.21	1.08	1.16	1.24	1.47	1.35	1.39	1.24	1.68
	CMV+ ISX	0.84	0.98	1.00	0.49	0.52	0.44	0.67	0.73	0.84	0.80	0.74
	MRTF+ ISX	1.33	1.27	1.22	0.72	0.65	0.67	0.99	1.01	1.09	1.25	1.15
CAVIN1 (Panel D)	CMV+ DMSO	0.76	1.16	1.13	0.93	1.06	1.01	0.97	1.03	0.91	1.09	1.00
	MRTF+ DMSO	1.17	1.39	1.18	1.44	1.51	1.57	1.21	1.23	1.27	1.18	1.50
	CMV+ ISX	0.71	0.83	0.69	0.58	0.68	0.65	0.61	0.71	0.63	0.90	0.77
	MRTF+ ISX	0.95	0.96	0.82	0.98	0.96	0.84	0.80	0.99	0.91	1.23	1.17
