# Supplementary material for: Similar regulatory mechanisms of caveolins and cavins by myocardin family coactivators in arterial and bladder smooth muscle
Source: PLoS One. 2017 May 25;12(5):e0176759. doi: 10.1371/journal.pone.0176759 (PMC5444588; doi:10.1371/journal.pone.0176759)
Supplement: S13 Table — (PDF) [file pone.0176759.s014.pdf]

S13 Table Data for Fig6 A to F

Panels	Targets	Log2 (mRNA reads) [raw data from GTeXPortal.org]										
Panel A	MYOCD	11.74	11.30	11.76	10.80	13.62	10.60	9.93	13.26	13.70	10.48	13.24
	CAV1	13.43	13.62	13.44	13.17	15.38	12.96	12.27	15.25	15.24	12.66	14.53
Panel B	MKL1	10.92	10.99	10.65	10.77	10.78	10.55	10.67	11.25	10.48	10.73	10.75
	CAV1	13.43	13.62	13.44	13.17	15.38	12.96	12.27	15.25	15.24	12.66	14.53
Panel C	MKL2	12.24	12.35	12.07	12.29	11.32	12.03	12.12	12.23	12.43	11.84	11.84
	CAV1	13.43	13.62	13.44	13.17	15.38	12.96	12.27	15.25	15.24	12.66	14.53
Panel D	MYOCD	11.74	11.30	11.76	10.80	13.62	10.60	9.93	13.26	13.70	10.48	13.24
	CAVIN1	14.57	14.11	14.49	14.14	16.21	13.59	12.74	15.53	15.63	13.68	15.27
Panel E	MKL1	10.92	10.99	10.65	10.77	10.78	10.55	10.67	11.25	10.48	10.73	10.75
	CAVIN1	14.57	14.11	14.49	14.14	16.21	13.59	12.74	15.53	15.63	13.68	15.27
Panel F	MKL2	12.24	12.35	12.07	12.29	11.32	12.03	12.12	12.23	12.43	11.84	11.84
	CAVIN1	14.57	14.11	14.49	14.14	16.21	13.59	12.74	15.53	15.63	13.68	15.27
